# Integrative analysis of blood transcriptome profiles in small-cell lung cancer patients for identification of novel chemotherapy resistance-related biomarkers

**DOI:** 10.3389/fimmu.2024.1338162

**Published:** 2024-06-18

**Authors:** Fang Yang, Jinhua Fan, Runxiang Yang, Yupeng Cun

**Affiliations:** ^1^ Department of the Second Medical Oncology, Third Affiliated Hospital of Kunming Medical University, Kunming, Yunnan, China; ^2^ Pediatric Research Institute, Ministry of Education Key Laboratory of Child Development and Disorders, Chongqing Key Laboratory of Translational Medical Research in Cognitive Development and Learning and Memory Disorders, Children’s Hospital of Chongqing Medical University, Chongqing, China; ^3^ National Clinical Research Center for Child Health and Disorders, China International Science and Technology Cooperation Base of Child Development and Critical Disorders, Children’s Hospital of Chongqing Medical University, Chongqing, China

**Keywords:** SCLC, chemotherapy resistance, primary resistance, acquired resistance, gene expression, network analysis

## Abstract

**Introduction:**

Chemoresistance constitutes a prevalent factor that significantly impacts thesurvival of patients undergoing treatment for smal-cell lung cancer (SCLC). Chemotherapy resistance in SCLC patients is generally classified as primary or acquired resistance, each governedby distinct mechanisms that remain inadequately researched.

**Methods:**

In this study, we performed transcriptome screening of peripheral blood plasma obtainedfrom 17 patients before and after receiving combined etoposide and platinum treatment. We firs testimated pseudo-single-cell analysis using xCell and ESTIMATE and identified differentially expressed genes (DEGs), then performed network analysis to discover key hub genes involved in chemotherapy resistance.

**Results:**

Our analysis showed a significant increase in class-switched memory B cell scores acrossboth chemotherapy resistance patterns, indicating their potential crucial role in mediatingresistance. Moreover, network analysis identifed *PRICKLE3*, *TNFSFI0*, *ACSLl* and *EP300* as potential contributors to primary resistance, with *SNWl*, *SENP2* and *SMNDCl* emerging assignificant factors in acquired resistance, providing valuable insights into chemotherapy resistancein SCLC.

**Discussion:**

These findings offer valuable insights for understanding chemotherapy resistance and related gene signatures in SCLC, which could help further biological validation studies.

## Introduction

Lung cancer ranks among the most prevalent malignancies worldwide, with the highest incidence and mortality rates among all tumor types. It is usually classified as non-small-cell lung cancer (NSCLC) and small-cell lung cancer (SCLC) (1). SCLC constitutes approximately 15% of lung cancer cases and is characterized by poor prognosis and high chemotherapy resistance (1). Approximately two-thirds of individuals with SCLC receive a diagnosis of extensive-stage SCLC (ES-SCLC), indicating that the disease has advanced beyond a field tolerable to radiation. As a result, treatment options for nearly 80% of patients are primarily limited to platinum-based chemotherapy (2). Based on genetic analysis, *TP53* and *RB1* have been identified as two frequently mutated genes in SCLC, exhibiting substantially lower subclonal diversity compared to NSCLC (3–5).

In recent decades, the combined etoposide-platinum (EP) regimen has become the standard first-line therapy for SCLC patients. Platinum-based doublet chemotherapy shows efficacy in 60% ~ 80% of ES-SCLC patients, but responses are typically short-lived (6). Many patients experience relapse within the first year of EP treatment, upon which a patient’s response to platinum-based chemotherapy is categorized. If relapse occurs during treatment, it is labeled as primary resistance (PR); within 90 days after treatment, it is referred to as acquired resistance (AR); and beyond 90 days, it is classified as platinum-sensitive (7). In SCLC, acquired resistance almost always leads to relapse, and there are no effective second-line treatments currently available. With the increase in the use of immune checkpoint inhibitors (ICIs) in cancer treatment, immune combination therapy has been approved as a first-line treatment for ES-SCLC. Nevertheless, this advancement has only resulted in a marginal extension of overall median survival time (12.3–13.0 months) (8, 9). Many studies have explored the molecular mechanisms underpinning chemotherapy resistance in SCLC, predominantly using cell lines and preclinical models. However, direct investigations of human SCLC trials remain rare, primarily due to limited datasets and insufficient patient follow-up regarding relapse time. RNA present in plasma harbors the potential to elucidate systemic responses to tumor progression and provide insights into the specific type of tissue from which tumors originate, particularly stratified by cancer type (10). This underscores the urgent need to accurately detect blood-based biomarkers of chemotherapy resistance in patients, thus providing a relatively non-invasive and real-time monitoring approach for assessing drug treatment responses. Gay et al. categorized SCLC patients into four subtypes based on expression profiles of transcriptional factors (*ASCL1*, *NEUROD1*, *POU2F3*, and *YAP1*), with each type exhibiting a distinct expression pattern and sensitivity to different therapeutic drugs (11). Based on these findings, we hypothesize that differences in transcriptome profiles between SCLC patients with primary and acquired resistance may be associated with chemotherapy resistance. Given the limited chemotherapy options available for SCLC patients, detecting chemotherapy resistance in SCLC remains an important challenge in clinical practice and management. As a result, a systematic investigation into how distinct genes and cell types contribute to chemotherapy resistance in SCLC is essential. Such research could yield valuable insights into potential targets for novel and effective treatment strategies.

In this study, we examined the potential mechanisms underlying primary and acquired resistance in SCLC patients. We collected 34 peripheral blood plasma samples from 17 SCLC patients before and after EP treatment, and performed gene expression screening and analysis to identify potential gene signatures and cell types associated with platinum-based chemotherapy resistance. Furthermore, weighted correlation network analysis (WGCNA) (12) was employed to construct a co-expression network of differentially expressed cancer-related genes, with minimum spanning tree (MST) analysis of the network then performed to identify key hub genes in chemotherapy resistance. We also performed pseudo-single-cell analysis of the samples using xCell and ESTIMATE to study the influence of immune cell composition on chemotherapy resistance.

## Materials and methods

### Patient sample collection

We collected 34 cryopreserved peripheral blood plasma samples from 17 SCLC patients before and after combined cisplatin and etoposide treatment at the Yunnan Cancer Hospital (China). All patient information is presented in **Supplementary Tables S1** and **S2**. The patients were divided into four groups based on their response to EP treatment: pre-treatment primary resistance (PR_pre), post-treatment primary resistance (PR_post), pre-treatment acquired resistance (AR_pre), and post-treatment acquired resistance (AR_post). All patients provided informed consent in accordance with the Helsinki Declaration. All research protocols were approved by the Ethics Committee of the Yunnan Cancer Hospital (KYLX202123).

### Gene expression screening, differential gene expression, and enrichment analysis

Total RNA from the plasma was extracted and purified using a Qiagen plasma kit (Cat. #217184, Qiagen) following the manufacturer’s standard instructions. RNA quantity and purity were then assessed using a NanoDrop ND-2000 spectrophotometer. Subsequently, the gene expression profiles of total RNA were screened using the Agilent Technologies platform. All experimental steps were conducted according to the standard instructions provided. Sequencing was conducted using the Agilent array, with samples from the same batch. The raw gene expression data were normalized using the quantile algorithm implemented in the limma package (v1.10.1) in R (4.1.3) (13).

Differentially expressed genes (DEGs) were estimated using the limma package based on a Benjamini-Hochberg-adjusted *p*-value ≤ 0.05 and |log2fold-change (FC)| > 0.3. Functional enrichment analysis of Kyoto Encyclopedia of Genes and Genomes (KEGG) pathways and Gene Ontology (GO) terms was conducted using KOBAS (http://kobas.cbi.pku.edu.cn/) (14). The gene interaction network associated with cancer-related KEGG pathways was visualized using Cytoscape (v3.8.0) (15).

### Immune cell type deconvolution

Immune cell type deconvolution was analyzed via xCell (v1.1.0) (16) and ESTIMATE (v1.0.13) (17). The ESTIMATE dataset calculated the pseudo-immune score of each sample as a novel biomarker of immune cell infiltration in cancer. The xCell datasets provided immune cell indices derived from the gene expression profiles of the samples. Pearson correlation analysis was used to compute correlation coefficients between immune infiltration scores and expression levels of hub genes. All correlation results were visualized using ggplot2 (v3.4.4) package.

### Construction of cancer-related gene co-expression network and identification of hub genes

WGCNA (v1.72.1) was used to construct a co-expression network and expression patterns of cancer pathway-related genes across the 34 samples. In the network, edge weights were filtered for edges with a threshold greater than 0.01, and only top the 30% of edges for each gene were included to generate the final network. Hub genes were identified through MST analysis of the final network, as applied in our former study (18). All networks were visualized with Cytoscape (v3.8.0).

### Gene set variation analysis of chemotherapy resistance genes

GSVA (v1.42.0) (19) was used to identify signaling pathways associated with candidate genes. Specifically, samples were divided into high and low expression groups based on the median expression values of the candidate genes and analyzed accordingly. A reference gene set for GSVA was selected with “h.all.v7.4.symbols.gmt”.

### Kaplan‐Meier survival analysis of candidate progression-associated genes

Samples were divided into high and low expression groups based on the median expression values of the candidate genes and analyzed. Survival curves, representing the time to tumor progression, were generated using the Kaplan-Meier method. To compare the survival curves between two groups, log-rank tests were applied in the survival package (v3.2.13) (20).

## Results

### Gene expression landscape and pseudo-single-cell profiles in SCLC patients before and after EP treatment

**Figure 1:**
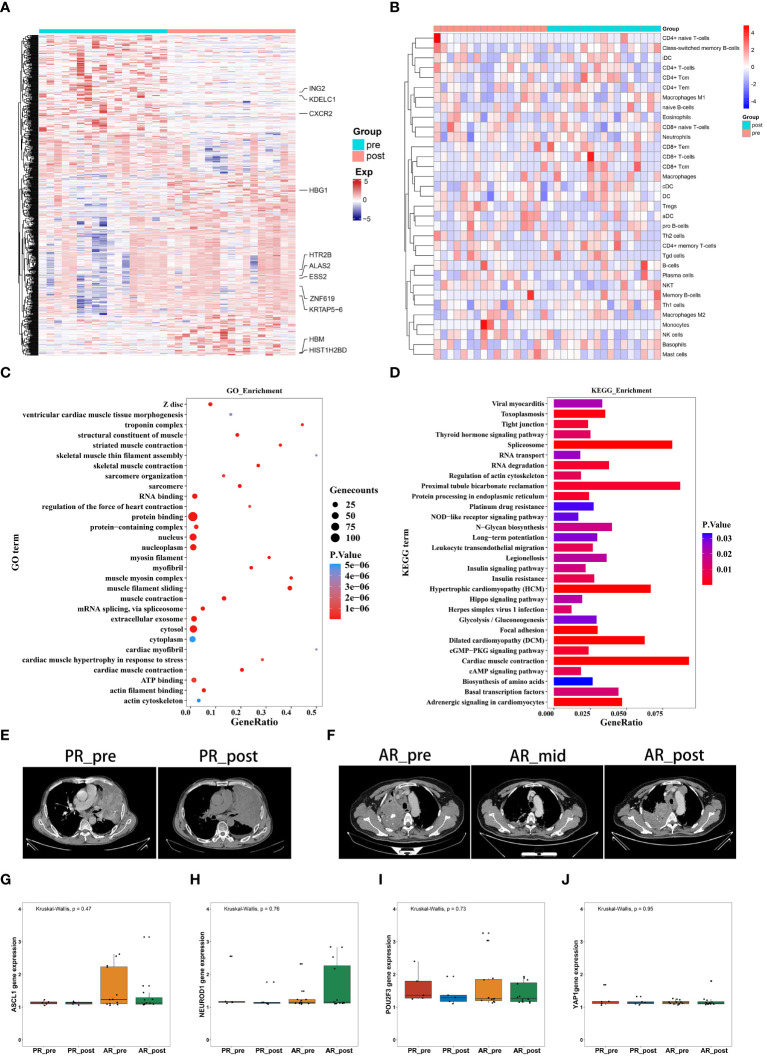
Gene expression profiles and pseudo-single-cell analysis of 17 patients with SCLC before and after combined cisplatin and etoposide treatment. **(A)** Heatmap analysis of DEGs between pre- and post-EP treatment groups. **(B)** Heatmap comparing expression levels of 64 cell components from xCell between pre- and post-EP treatment groups. **(C)** GO functional enrichment analysis, showing 30 most enriched GO terms. **(D)** KEGG enrichment analysis, showing 30 most enriched KEGG pathways. **(E)** Radiographic images of patients with acquired resistance before, during, and after EP treatment. **(F)** Radiographic images of patients with primary resistance before and after EP treatment. **(G-J)** Expression levels of *ASCL1*, *NEUROD1*, *POU2F3*, and *YAP1* in PR_pre, PR_post, AR_pre, and AR_post groups.

We collected 34 peripheral blood plasma samples from 17 SCLC patients before and after EP treatment for gene expression array screening (**Supplementary Table S2**). The SCLC patients before and after EP treatment were designated as the pre-group and post-group, respectively. All analysis workflows are depicted in **Supplementary Figure S1**. Paired DEG analysis of the pre- and post-treatment patients was performed using the limma package, with significance threshold set at |log2FC| > 0.3 and *p* ≤ 0.05. A total of 594 DEGs were identified, including 336 up-regulated genes and 258 down-regulated genes. Genes with |log2FC| ≥ 1 are annotated in **Figure 1A** and **Supplementary Figure S2A, B**. To assess tumor pseudo-single-cell changes between the pre- and post-groups, we employed xCell to compute cell scores for 64 distinct cell types (**Figure 1B**). The xCell results showed no significant differences in immune, stromal, and microenvironment scores between the pre- and post-groups, although there were observable declining trends in variation (**Supplementary Figure S2C–E**). ESTIMATE was also applied to compare changes in the blood microenvironment between the pre- and post-groups, showing results in line with the xCell findings (**Supplementary Figure S2F–H**). The immune cell scores estimated using xCell showed no significant differences among immune cells, including CD4+ T memory cells, natural killer (NK) T cells, and regulatory T cells, but exhibited a decreasing trend in variation (**Supplementary Figure S2–I**).

GO term enrichment analysis demonstrated that genes influenced by EP treatment were predominantly associated with “protein binding”, “RNA binding”, and “nucleus” (**Figure 1C**). Among the genes influenced by EP treatment, *EP300* is widely expressed and regulates several essential biological processes, including proliferation and differentiation, through its paralogous domains, with mutations in *EP300* known to accelerate tumor development in SCLC mouse models (21). KEGG enrichment analysis revealed that genes influenced by EP treatment were highly associated with various pathways, including “RNA degradation”, “Platinum drug resistance”, and “IL-17 signaling pathway” (**Figure 1D**). Elevated levels of IL-17 in hepatocellular carcinoma, colorectal cancer, and NSCLC are indicative of poor prognosis (22, 23). Furthermore, IL-17 promotes angiogenesis by up-regulating various post-angiogenic factors in tumor cells and fibroblasts and contributes to tumor resistance against VEGF therapy (24, 25). Thus, the KEGG and GO enrichment analyses revealed that the DEGs were associated with drug resistance, immune response, and epigenetic regulation.

Patients underwent CT imaging assessment every two treatment cycles, strictly following RECIST1.1 criteria for efficacy evaluation. Patients who experienced partial remission followed by progression were defined as having acquired resistance, while disease progression occurring after stability or initial assessment was defined as primary resistance. Based on these criteria, we classified the 17 patients into two distinct groups. Notably, five patients exhibited minimal changes in tumor size before and after treatment, indicating primary resistance (**Figure 1E**), while 12 patients exhibited tumor relapse after EP treatment, with the emergence of new tumors within three months, indicating acquired resistance (**Figure 1F**). We compared the expression levels of transcription factors (*ASCL1*, *NEUROD1*, *POU2F3*, and *YAP1*) in the four pre-defined subtypes of SCLC among these patient groups (**Figures 1G–J**). Although group differences in the expression levels of transcription factors did not reach statistical significance, possibly due to the small sample size, the primary resistance patients exhibited low NEUROD1 and ASCL1 expression before treatment, while the acquired resistance patients exhibited low NEUROD1 expression and high ASCL1 expression before treatment, with contrasting expression patterns after treatment.

Overall, these findings highlight the distinct expression patterns of NEUROD1 and ASCL1 in primary and acquired resistance patients before and after EP treatment, suggesting a potential association with primary and acquired resistance.

### Gene signature variation between primary and acquired resistance in pre-patients

**Figure 2:**
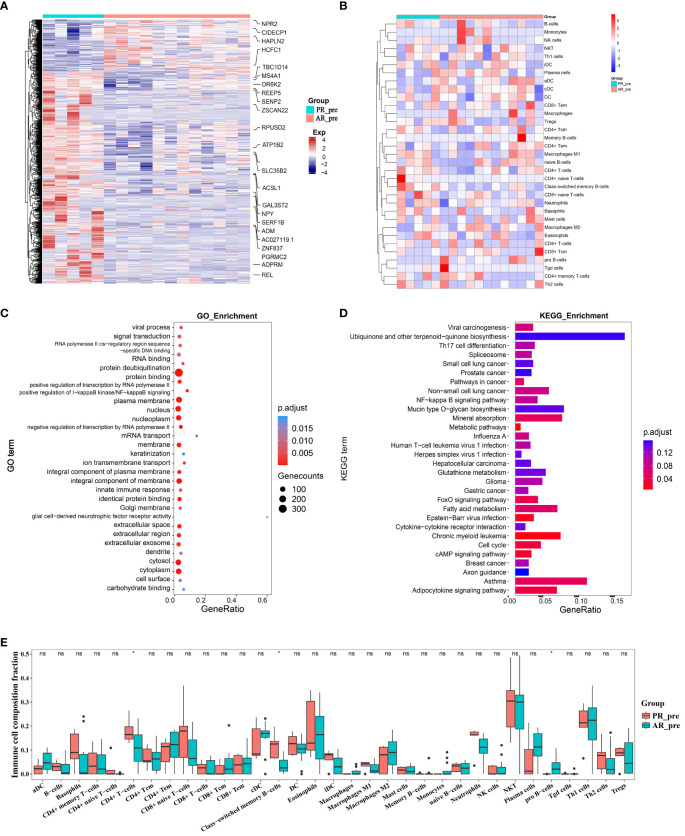
Changes in expression between primary and acquired resistance patients before cisplatin and etoposide treatment. **(A)** Heatmap analysis of DEGs between PR_pre and AR_pre groups. **(B)** Heatmap comparing expression levels of 64 cell components from xCell between PR_pre and AR_pre groups. **(C)** GO functional enrichment analysis, showing 30 most enriched GO terms. **(D)** KEGG enrichment analysis, showing 30 most enriched KEGG pathways. **(E)** Boxplot showing xCell enrichment scores of 33 immune cells between PR_pre and AR_pre groups, where * indicates p-value < 0.05 and ns indicates not significant.

To identify possible genes involved in primary resistance, we compared the transcriptome profiles of the pre-treatment primary resistance group (PR_pre, five cases) with the pre-treatment acquired resistance group (AR_pre, 12 cases). Genes with | log2FC | > 0.3 and *p ≤* 0.05 were identified as DEGs. Volcano plots and heatmaps were generated to visualize the DEGs in the PR_pre group versus the AR_pre group. In total, 664 DEGs were identified, including 130 up-regulated genes and 534 down-regulated genes (**Figure 2A**, **Supplementary Figure S3A**). Among the down-regulated genes, *ACSL1* encodes the long-chain fatty acid CoA synthetase and is associated with an increased risk of hepatocellular carcinoma (26). The DEGs displayed varying expression patterns between primary and acquired resistance (**Supplementary Figure S3B**). For instance, *TRPC3* showed the opposite pattern in the PR_pre and AR_post groups but demonstrated a similar expression pattern in the AP_pre and AR_post groups. *TRPC3* is a high-calcium permeable cation channel that regulates calcium-dependent signaling pathways involved in the proliferation and metastasis of ovarian cancer (27). KEGG and GO enrichment analysis revealed that the DEGs between the PR_pre and AR_post groups were predominantly associated with “protein binding”, “integral component of membrane”, and “plasma membrane” (**Figure 2C**), as well as the “NF-κB signaling pathway”, “Cell cycle”, and “Th17 cell differentiation” (**Figure 2D**). Activation and interaction between *STAT3* and the NF-κB signaling pathway play a crucial communication role between cancer cells and inflammatory cells. This interaction regulates various aspects of tumor biology, including tumor initiation, resistance of malignant cells to apoptosis-based tumor surveillance, tumor angiogenesis, and invasive capabilities (28).

We further compared immune and stromal scores between the two groups using xCell and ESTIMATE analysis, with no significant differences observed (**Figure 2B**, **Supplementary Figures S3C–H**). However, compared to the AR_pre group, the PR_pre group exhibited an increasing trend in class-switched memory B cells and CD4+ T cells among immune cell types, as well as a decreasing trend in pro B cells (**Figure 2E**). Class-switched memory B cells are a crucial component of reactive humoral immunity, ensuring the rapid production of high-affinity antigen-specific antibodies during antigen challenge. Smokers have a higher proportion of class-switched memory B cells compared to ex-smokers and never-smokers, regardless of whether they have chronic obstructive pulmonary disease (COPD) (29). CD4+ T cells can target tumor cells through various mechanisms, either by directly eliminating tumor cells through cytotoxicity or by indirectly eliminating tumor cells by regulating the tumor microenvironment (30). Furthermore, within secondary lymphoid organs, CD4+ T cells can enhance the intensity and quality of B cell and cytotoxic T lymphocyte responses (31). These findings suggest that patients with primary resistance exhibit a heightened immune response. These findings suggest that primary resistance is closely associated with cellular immunity and may be influenced by immune responses.

### Network analysis discovered key hub genes in primary resistance

**Figure 3:**
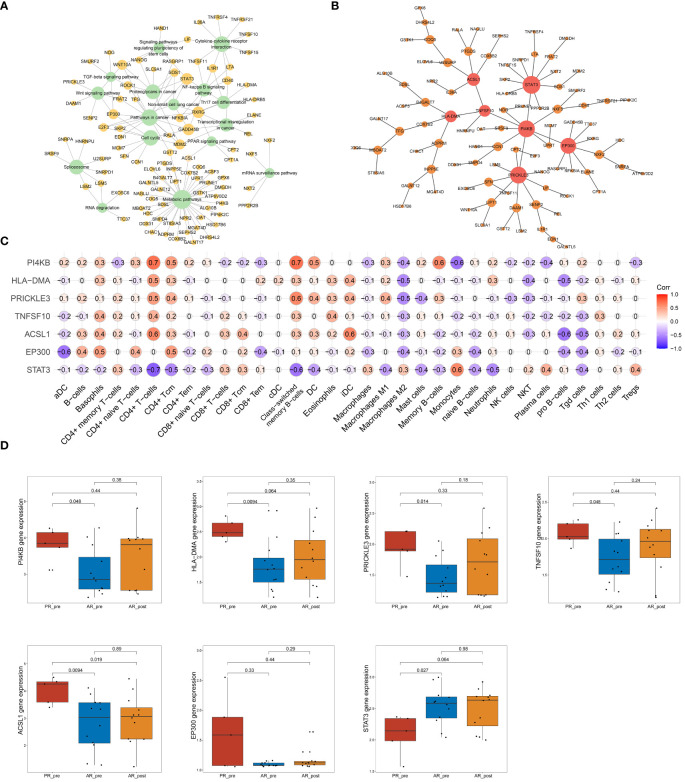
Network analysis of key hub genes in primary resistance. **(A)** Network plot showing DEGs between PR_pre and AR_pre groups associated with cancer-related KEGG pathways. **(B)** Network depicting co-expressed DEGs between PR_pre and AR_pre groups within cancer-associated KEGG pathways following WCGNA and MST analysis. **(C)** Correlation analysis between *PI4KB*, *HLA*-*DMA*, *PRICKLE3*, *TNFSF10*, *ACSL1*, *EP300*, and *STAT3* expression and immune cells. **(D)** *PI4KB*, *HLA-DMA*, *PRICKLE3*, *TNFSF10*, *ACSL1*, *EP300*, and *STAT3* expression between PR_pre, AR_pre and AR_post groups.

To further identify key genes associated with primary resistance in SCLC, we performed KEGG pathway enrichment analysis of DEGs between the PR_pre and AR_pre groups (**Figure 3A**). A co-expression network consisting of 95 cancer-related genes was constructed based on WGCNA. After filtering out the top 30% of edges, the final network contained 95 nodes with 1 499 edges (**Supplementary Figure S4A**). Furthermore, MST analysis of the network identified seven hub genes (node degree > 4), i.e., *PI4KB*, *HLA-DMA*, *PRICKLE3*, *TNFSF10*, *ACSL1*, *EP300*, and *STAT3* (**Figure 3B**). Subsequently, correlation analysis was performed between the seven hub genes and cell scores derived from the xCell. These results showed a strong correlation between these genes and immune cell types, particularly CD4+ T cells, which exhibited an increasing trend in the PR_pre group (**Figure 3C**). Therefore, these genes may play a role in immune suppression among patients with primary resistance. Comparative analysis of the seven hub genes among the PR_pre, AR_pre, and AR_post groups showed that *STAT3* was notably down-regulated in the PR_pre group, whereas *PI4KB*, *HLA-DMA*, *PRICKLE3*, *TNFSF10*, *ACSL1*, and *EP300* were up-regulated in the PR_pre group compared to the AR_pre group. Interestingly, *EP300* did not show a significant difference in expression between the AR_pre and AR_post groups but displayed a nearly 1.5-fold higher expression in the Pre_post group (**Figure 3D**). Compared to the AR_pre group, *PI4KB*, *HLA-DMA*, *PRICKLE3*, and *TNFSF10* showed a similar increased trend in the AR_post and PR_pre groups (**Figure 3D**).

**Figure 4:**
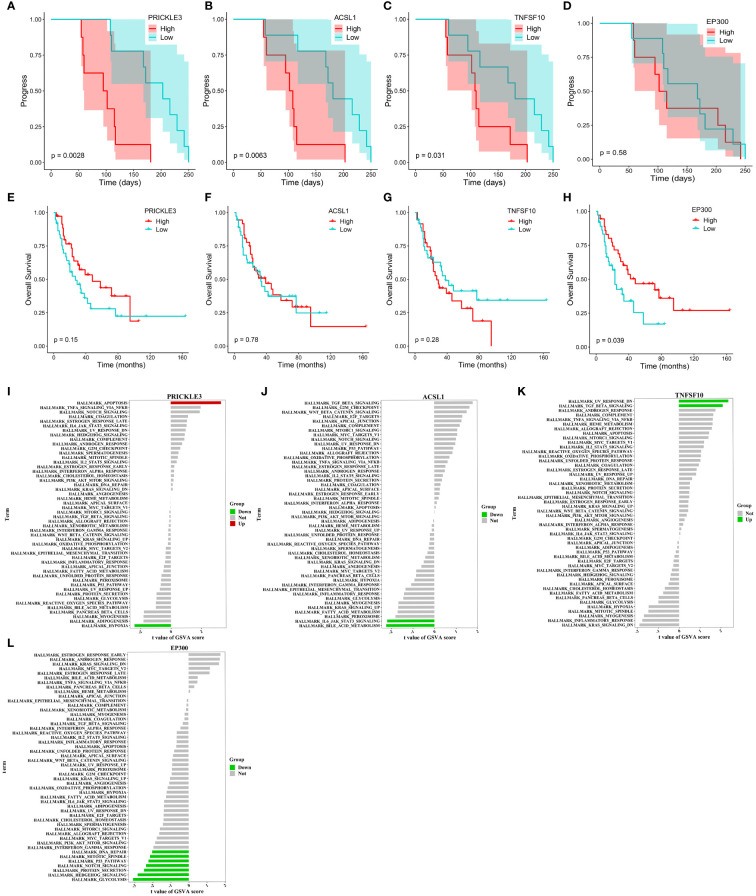
Progression, survival and GSVA analysis of hub genes in primary resistance. **(A-D)** Kaplan-Meier curve of *PRICKLE3*, *ACSL1*, *TNFSF10* and *EP300* with progression time. **(E-H)** Kaplan-Meier curve of *PRICKLE3*, *ACSL1*, *TNFSF10* and *EP300* with overall survival time. **(I-L)** GSVA of PRICKLE3, ACSL1, TNFSF10 and EP300.

We tracked and analyzed the relationship between the seven hub genes and disease progression for each patient and revealed a significant correlation between *PRICKLE3*, *TNFSF10*, and *ACSL1* and disease progression (**Figures 4A–D**, **Supplementary Figure S4B–D**). We collected gene expression and clinical information of tumor tissues obtained from 73 patients in George et al. (3), which revealed a significant correlation between the *EP300* gene and survival prognosis (**Figures 4E–H**). To predict the functions of the seven genes, we conducted GSVA based on gene sets from the MSigDB database v7.1 (**Figures 4I–L**, **Supplementary Figure S4E–G**). Results showed a negative correlation between *ACSL1* expression and the pathway score of “IL6 JAK *STAT3* SIGNALING” (**Figure 4I**), a positive correlation between *TNFSF10* expression and the pathway score of “TGF BETA SIGNALING” (**Figure 4J**), a positive correlation between *PRICKLE3* expression and the pathway score of “APOPTOSIS” (**Figure 4K**), and a negative correlation between *EP300* expression and the pathway scores of “GLYCOLYSIS”, “NOTCH SIGNALING”, and “P53 PATHWAY” (**Figure 4L**), suggesting these genes may play a role in immune regulation and cellular apoptosis. Previous research has demonstrated that the tumor necrosis factor (TNF) superfamily, comprised of 19 cytokines, triggers a wide range of cellular responses via homologous receptors and two principal signaling pathways. These pathways encompass the initiation of apoptosis through caspase activation, along with the stimulation of inflammation and cellular differentiation programs (32, 33). In conclusion, these results suggest that *PRICKLE3*, *ACSL1*, *TNFSF10*, and *EP300* may induce primary resistance in SCLC patients by modulating immune function and cellular apoptosis.

### Gene signature variation before and after EP treatment in acquired resistance patients

**Figure 5:**
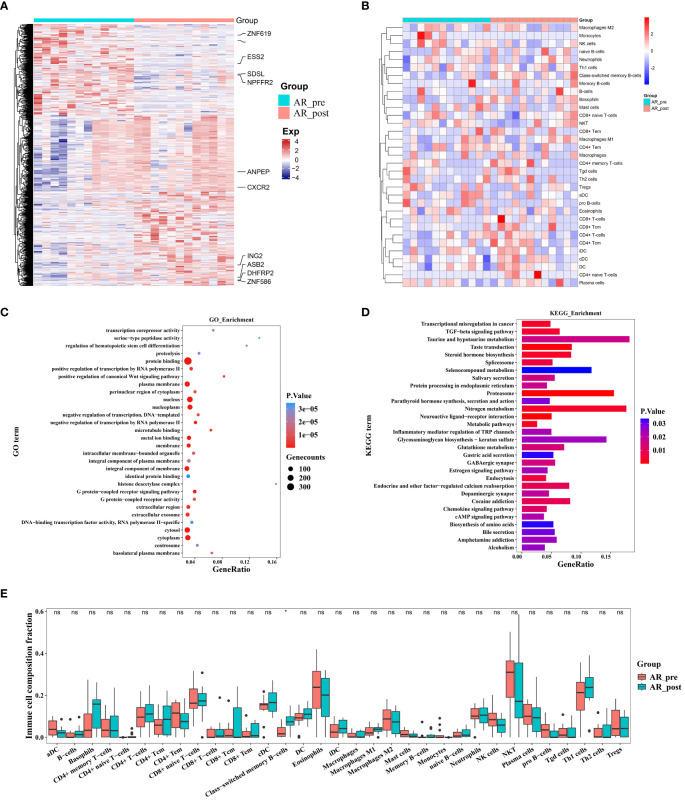
Changes in expression in acquired resistance patients before and after cisplatin and etoposide treatment. **(A)** Heatmap analysis of DEGs between AR_pre and AR_post groups. **(B)** Heatmap comparing expression levels of 64 cell components from xCell between AR_pre and AR_post groups. **(C)** GO functional enrichment analysis, showing 30 most enriched GO terms. **(D)** KEGG enrichment analysis, showing 30 most enriched KEGG pathways. **(E)** Boxplot showing xCELL enrichment scores of 33 immune cells between AR_pre and AR_post groups, where * indicates p-value < 0.05 and ns indicates not significant.

To elucidate the molecular mechanisms of acquired resistance to EP treatment, we compared the transcriptome profiles of acquired resistance patients before and after treatment. Paired differential transcriptional expression analysis was performed on the AR_pre and AR_post groups using limma, and gene signatures were selected based on genes with |log2FC| > 0.3 and *p ≤* 0.05. In total, 745 DEGs were identified, including 486 up-regulated genes and 259 down-regulated genes, with genes showing |log2FC| ≥ 1 annotated and plotted (**Figure 5A**, **Supplementary Figure S5A**). We compared the expression patterns of the DEGs across the four groups. The gene expression profiles showed variation in the AR_post and PR_pre groups, further confirming that the mechanisms underlying primary and acquired resistance are not identical (**Supplementary Figure S5B**). For example, *ESS2* exhibited opposite expression levels in the PR_pre and AR_post groups. *ESS2*, also known as *DGCR14*, is a transcriptional co-regulator in CD4+ T cells and plays a critical role in thymocyte survival through the *MYC* and IL-7 signaling pathways (34). GO enrichment analysis revealed that the genes influenced by EP treatment were predominantly associated with “protein binding”, “nucleus”, and “plasma membrane” (**Figure 5C**). KEGG enrichment analysis revealed that resistance-biased genes were highly associated with “Proteasome”, “Nitrogen metabolism”, and “TGF-beta signaling pathway” (**Figure 5D**). The transforming growth factor-beta (TGF-β) signaling pathway functions to suppress tumors in healthy cells and early-stage cancer cells by inducing cell cycle arrest and apoptosis, but can promote tumor progression, including metastasis and chemotherapy resistance, when activated in advanced-stage cancer (35).

We next compared the immune and stromal scores between the AR_post and AR_pre groups based on xCell and ESTIMATE analysis (**Figure 5B**, **Supplementary Figure S5C–H**). Results revealed that the AR_post group exhibited a significant increase in class-switched memory B cells among immune cell types compared to the AR_pre group (**Figure 5E**). We mainly focused on the molecular profiles of acquired resistance, excluding SCLC patients with primary resistance, and discovered that acquired resistance may be exerted through pathways involving immune regulation, metabolism, and protein degradation.

### Network analysis of key hub genes in acquired resistance

**Figure 6:**
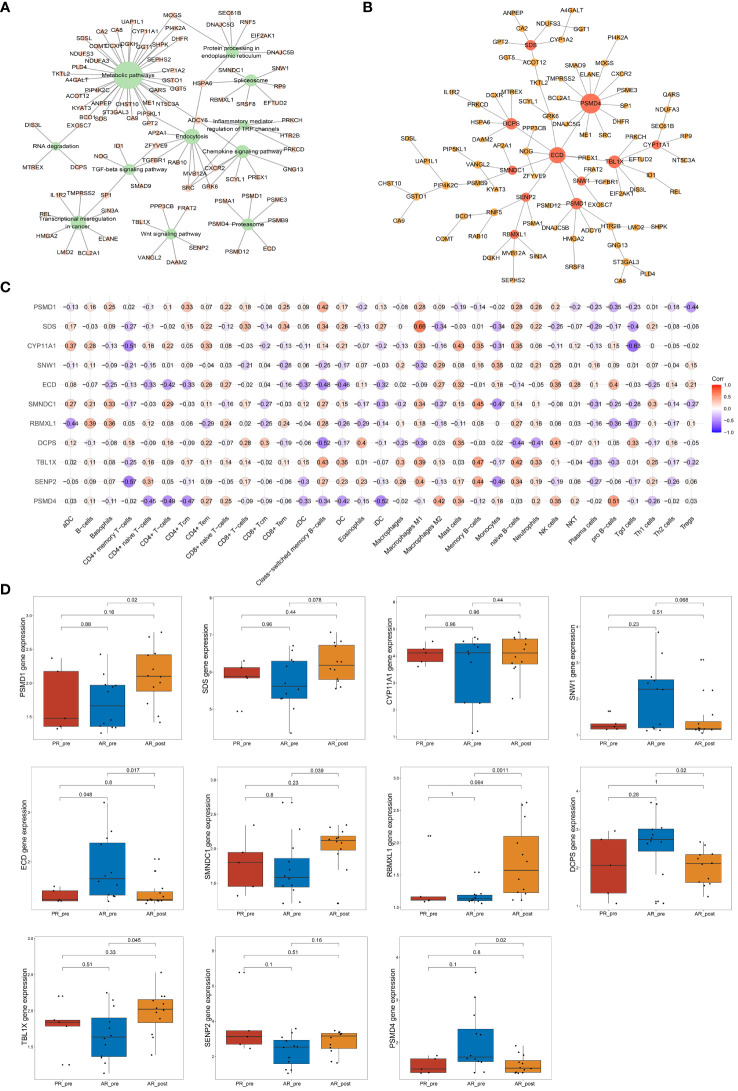
Network analysis of key hub genes in acquired resistance. **(A)** Network plot showing DEGs between AR_pre and AR_post groups associated with cancer-related KEGG pathways. **(B)** Network depicting co-expressed DEGs between AR_pre and AR_post groups within cancer-associated KEGG pathways following WCGNA and MST analysis. **(C)** Correlation analysis between *PSMD4*, *SENP2*, *TBL1X*, *DCPS*, *PBMXL1*, *SMNDC1*, *ECD*, *SNW1*, *CYP11A1*, *SDS*, and *PSMD1* expression and immune cells. **(D)** *PSMD4*, *SENP2*, *TBL1X*, *DCPS*, *PBMXL1*, *SMNDC1*, *ECD*, *SNW1*, *CYP11A1*, *SDS*, and *PSMD1* expression between PR_pre, AR_pre and AR_post groups.

To further identify key genes associated with acquired resistance in SCLC, we identified DEGs between the AR_pre and AR_post groups and performed KEGG pathway enrichment analysis of cancer-related pathways (**Figure 6A**). The expression matrix of the 92 genes enriched in these pathways was used to construct a co-expression network via WGCNA. After filtering out the top 30% of edges of each node, the core constructed network contained 92 nodes and 2 582 edges (**Supplementary Figure S6A**). In total, 11 hub genes (node degree > 4) were identified via MST analysis including *SENP2*, *TBL1X*, *DCPS*, *PBMXL1*, *SMNDC1*, *ECD*, *SNW1*, *CYP11A1*, *SDS*, *PSMD4* and *PSMD1* (**Figure 6B**). Subsequently, correlation analysis was performed between the 11 hub genes and cell scores derived from the xCell, which showed a strong positive correlation between *SDS* and macrophage M1 cells, and *SNW1* and megakaryocyte cells (**Figure 6C**). *SNW1* is a nuclear matrix-associated coactivator that can interact with the vitamin D receptor to mediate transcription and RNA splicing (36) and is essential for TGF-β1-induced epithelial-mesenchymal transition and invasion (37). These findings suggest that both *SNW1* and megakaryocyte cells play important roles in tumor invasion, consistent with the strong correlation observed between *SNW1* and megakaryocyte cells in our study. We further conducted a comparative analysis of the expression patterns of the 11 genes among the AR_pre, AR_post, and PR_pre groups. Notably, *SENP2*, *PBMXL1*, *TBL1X*, *SMNDC1*, *SDS*, *PSMD1*, and *CYP11A1* were up-regulated, while *ECD*, *DCPS*, and *SNW1* showed down-regulated expression in the AR_post group compared to the AR_pre group. Although there was no significant difference in the expression levels of *CYP11A1* and *SENP2* between the two groups, an upward trend was observed in the AR_post group (**Figure 6D**). *SDS*, *ECD*, *CYP11A1*, *DCPS*, *TBL1X*, and *SENP2* showed similar trends in the AR_post and PR_pre groups compared to the AR_pre group (**Figure 6D**).

**Figure 7:**
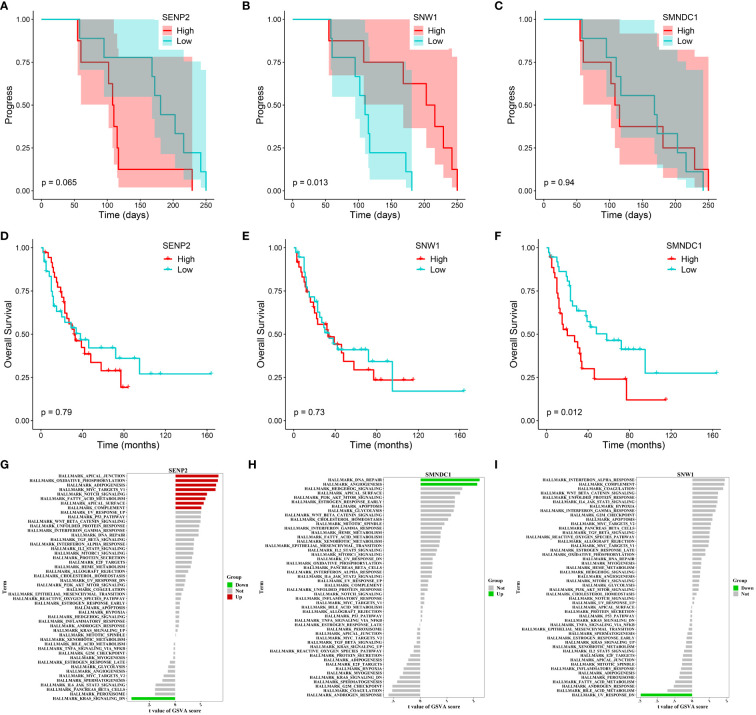
Progression, survival and GSVA analysis of hub genes in acquired resistance. **(A-C)** Kaplan-Meier curve of *SENP2*, *SNW1*, and *SMNDC1* with progression time. **(D-F)** Kaplan-Meier curve of *SENP2*, *SNW1*, and *SMNDC1* with overall survival time. **(G-I)** GSVA of *SENP2*, *SNW1*, and *SMNDC1*.

We also tracked and analyzed the relationship between the 11 hub genes and disease progression for each patient and demonstrated a significant correlation between *SNW1* and *SENP2* and disease progression (**Figures 7A–C**, **Supplementary Figure S6B–I**). Furthermore, in the dataset obtained from George et al., a significant correlation was observed between the *SMNDC1* gene and survival prognosis (**Figures 7D–F**). To predict the functions of the 11 genes, we conducted GSVA based on gene sets from the MSigDB database v7.1 (**Figures 7G–I**, **Supplementary Figure S7A–H**). Results showed a negative correlation between *SNW1* expression and the pathway score of “UV_RESPONSE_DN” (**Figure 7G**) and positive correlations between *SENP2* expression and the pathway scores of “OXIDATIVE_PHOSPHORYLATION”, “MYC_TARGETS_V1”, and “NOTCH_SIGNALING” (**Figure 7H**), a negative correlation between *SMNDC1* expression and the pathway score of “UV_RESPONSE_DN” (**Figure 7I**). Studies have shown that *SENP2*, *DCUN1D1*, *DVL3*, and *UBXN7* participate in a small protein-protein interaction network. Knockout of any of these four genes results in suppressed growth of lung squamous cell carcinoma with 3q26–29 amplification (36). Furthermore, knockdown of *SENP2* results in significant inhibition of cell growth (38). The Notch signaling pathway and *MYC* targets play broad roles in the promotion or inhibition of proliferation and cell death (39, 40), related to the regulatory role of *SENP2* in tumor growth.

In summary, these findings suggest that *SNW1*, *SENP2* and *SMNDC1* may regulate acquired resistance in SCLC patients by modulating the Notch and *MYC* signaling pathways.

## Discussion

SCLC is a highly invasive neuroendocrine tumor characterized by rapid cell division, a high proportion of actively proliferating cells, and early dissemination to distant sites in the body (41). Earlier studies have shown that the expression of *ASCL1* declines in patients with SCLC and in SCLC cells following cisplatin treatment, particularly in cases with acquired chemoresistance, in comparison to untreated samples (42). In recent years, the consensus definition of SCLC subtypes has transitioned from classical/variant classification to neuroendocrine (NE)/non-neuroendocrine (non-NE) classification, with further subgroup categorization based on specific transcription factors, i.e., NE transcription factors *ASCL1* and/or *NEUROD1* (43, 44), non-NE variants associated with *POU2F3*, and a hypothetical fourth subtype driven by the transcription factor *YAP1* (45). In the current study, we observed that primary resistance patients displayed decreased expression of *ASCL1* and *NEUROD1*, while acquired resistance patients exhibited increased expression of *NEUROD1* and decreased expression of *ASCL1* following cisplatin and etoposide treatment. These findings suggest that primary and acquired resistance patients belong to distinct SCLC transcriptional subtypes, characterized by different mechanisms of drug resistance, which need further research to enhance our understanding of their unique features.

SCLC is closely associated with the tumor microenvironment, typified by a high mutational burden, and induces paraneoplastic autoimmune syndromes in up to a third of patients. In principle, these attributes suggest that SCLC could potentially exhibit a positive response to immune checkpoint blockade (46). However, the combined use of chemotherapy and ICIs targeting programmed cell death ligand 1 (PD-L1), such as durvalumab and atezolizumab, has yielded only modest improvement in survival outcomes for SCLC patients (9, 47). Similarly, the use of the programmed cell death protein 1 (PD-1) checkpoint blockade antibody nivolumab in SCLC patients has also yielded disappointing results (48). Thus, there is a critical need to establish a comprehensive understanding of the interactions between the immune system and SCLC. Previous studies have shown that genetically modified mouse models lacking NK cells display a heightened tumor burden under SCLC metastasis conditions (49). In other words, NK cells possess the ability to activate interleukin-15 (IL-15) to mitigate tumor mutational burden in SCLC models, indicating that targeting NK cells within the SCLC microenvironment may provoke a specific response. Here, we found a significant increase in class-switched memory B cells in both primary and acquired resistance patients after EP treatment. Prior studies have also reported that class-switched memory B cell abundance is higher in poor prognosis subtypes of hepatocellular carcinoma and contributes to the prevalence of immune-related adverse events (IRAs) associated with ICIs (50). Our findings suggest that class-switched memory B cells may play a role in platinum treatment resistance in SCLC patients, which warrants further research.

Certain genes have been identified as significant players of platinum resistance in SCLC patients based on their mutation status. Among these genes, *COL11A1*, which encodes the α1 chain of type XI collagen, ranks as the third most mutated gene, following *TP53* and *RB1* (42). As a critical component of the extracellular matrix, dysregulation of *COL11A1* plays a key role in mediating platinum-based chemotherapy resistance (51, 52). Leslie et al. detected somatic copy number changes in *ABCC1* in recurrent SCLC, a membrane protein of the ATP-binding cassette family capable of effluxing both physiological compounds and drugs from the cytoplasm, thus suggesting a possible role in driving chemotherapy resistance (53). Approximately 20% of SCLC cases exhibit amplification of the *MYC* family oncogenes *MYCL1*, *MYCN*, and *MYC*, which is associated with poorer survival outcomes (3, 54). Cell lines derived from patients under platinum treatment also show approximately three times higher amplification rates of *MYC*, *MYCN*, and *MYCL* compared to those without platinum treatment (55). Studies using genetically engineered mouse models with combined *Rb1* and *Trp53* loss, coupled with *MYCL* or *MYCN* overexpression, have also demonstrated the involvement of *MYCL* and *MYCN* in platinum resistance (56).

In this study, we identified several genes related to primary resistance, specifically *PRICKLE3*, *TNFSF10*, *ACSL1* and *EP300*, which exhibited significant correlation with SCLC progression. *PRICKLE3* encodes a highly conserved protein enriched in mitochondria, with its *Drosophila* and *Xenopus* homologs involved in planar cell polarity and visual function (57). *PRICKLE3* is also associated with susceptibility alleles and mutations in Leber’s hereditary optic neuropathy (58, 59). The *TNFSF10* pathway constitutes an important component of the innate host anti-tumor immune surveillance mechanism, engaging in the selective activation of extrinsic cell death pathways in cancer cells (33). Recent studies have demonstrated significant up-regulation of *ACSL1* and *ACSL3* protein levels in highly metastatic ovarian cancer cell lines, with gene expression associated with increased metastatic capacity and poor survival prognosis (60). However, despite extensive research on *PRICKLE3*, *TNFSF10*, *ACSL1* and *EP300* in relation to cancer, immunity, and metastasis, their precise involvement in primary resistance mechanisms in SCLC remains unclear and requires further study. Similarly, the key genes *SNW1*, *SENP2* and *SMNDC1* identified in this study in the context of acquired resistance, have previously been implicated in cancer cell proliferation, death, and metastasis, although their specific role in regulating platinum resistance remains unknown. Consequently, our future research will focus on investigating and validating the connections among *PRICKLE3*, *TNFSF10*, *ACSL1*, *EP300*, *SNW1*, *SENP2* and *SMNDC1* in relation to SCLC resistance.

In conclusion, we conducted integrative bioinformatics analysis of the gene expression profiles of 34 blood samples collected from 17 SCLC patients before and after EP treatment. Our results suggest the potential significance of class-switched memory B cells in both primary and acquired resistance mechanisms. Notably, we identified *PRICKLE3*, *TNFSF10*, *ACSL1* and *EP300* as potential key gene regulators associated with primary resistance, and *SNW1*, *SENP2* and *SMNDC1* as key gene regulators associated with acquired resistance. These findings offer valuable insights for understanding chemotherapy resistance and related gene signatures in SCLC, which could help further biological validation studies.

## Data availability statement

The original contributions presented in the study are included in the article/**Supplementary Material**, further inquiries can be directed to the corresponding author/s.

## Ethics statement

The studies involving humans were approved by Ethics Committee of the Yunnan Cancer Hospital (KYLX202123). The studies were conducted in accordance with the local legislation and institutional requirements. The participants provided their written informed consent to participate in this study. Written informed consent was obtained from the individual(s) for the publication of any potentially identifiable images or data included in this article.

## Author contributions

FY: Writing – original draft, Data curation, Resources. JF: Methodology, Writing – original draft, Data curation, Formal analysis, Resources. RY: Writing – review & editing, Conceptualization, Funding acquisition, Methodology, Supervision. YC: Conceptualization, Writing – review & editing, Funding acquisition, Methodology, Supervision.
