# Supplementary material for: Integrative analysis of blood transcriptome profiles in small-cell lung cancer patients for identification of novel chemotherapy resistance-related biomarkers
Source: Front Immunol. 2024 Jun 18;15:1338162. doi: 10.3389/fimmu.2024.1338162 (PMC11217175; doi:10.3389/fimmu.2024.1338162)
Supplement: Supplementary file 1 [file Presentation_1.pdf]

Supplementary material

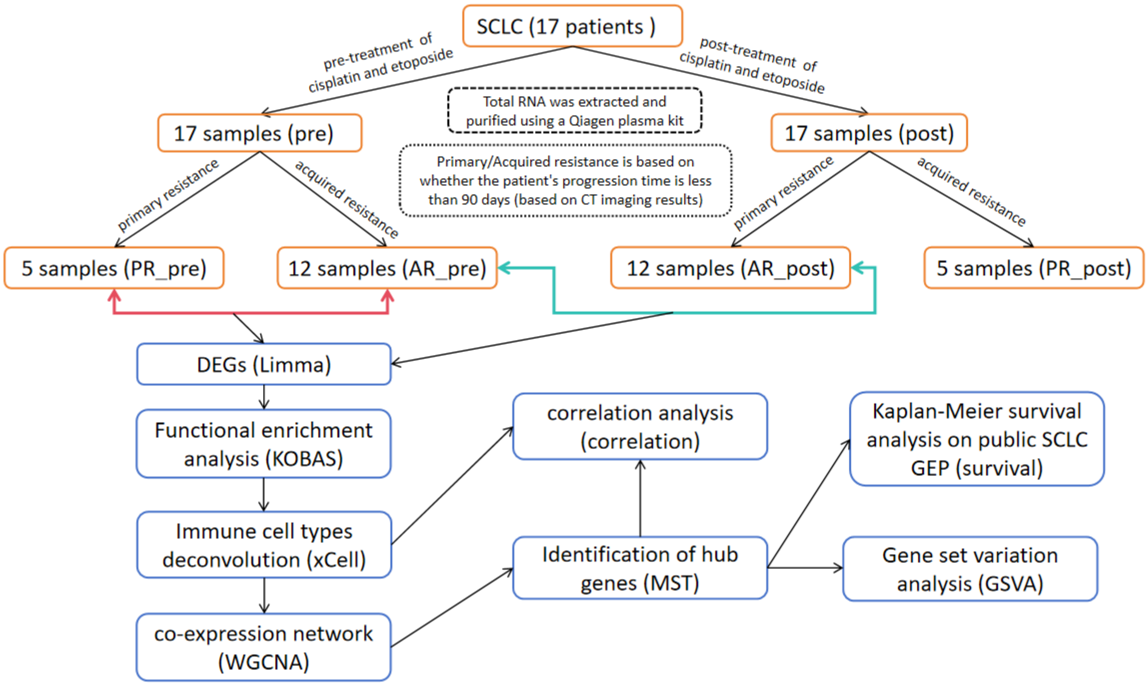

Supplementary Figure S1. The technical roadmap of the entire document.

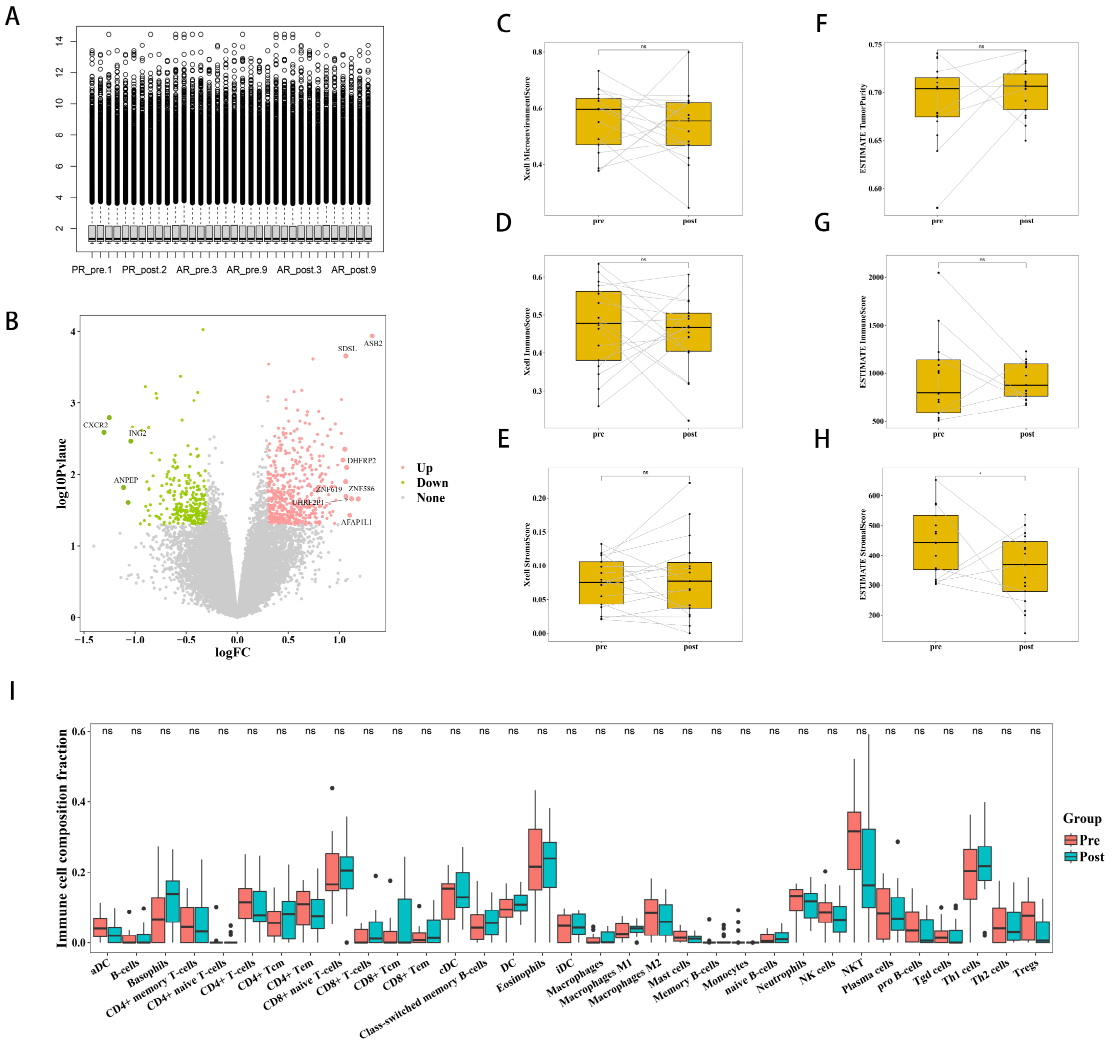

Supplementary Figure S2. (A) Histogram of gene expression for patients after batch correction. (B) Volcano plots showing differences between pre- and post-EP treatment groups. (C-E) Boxplot showing xCell enrichment microenvironment, stromal, and immune scores between pre- and post-EP treatment groups. (F-G) Boxplot showing ESTIMATE enrichment tumor purity, immune, and stromal scores between pre- and post-EP treatment groups. (I) Boxplot showing xCell enrichment scores of 33 immune cells between pre- and post-EP treatment groups. * p-value < 0.05 and ns indicates not significant.

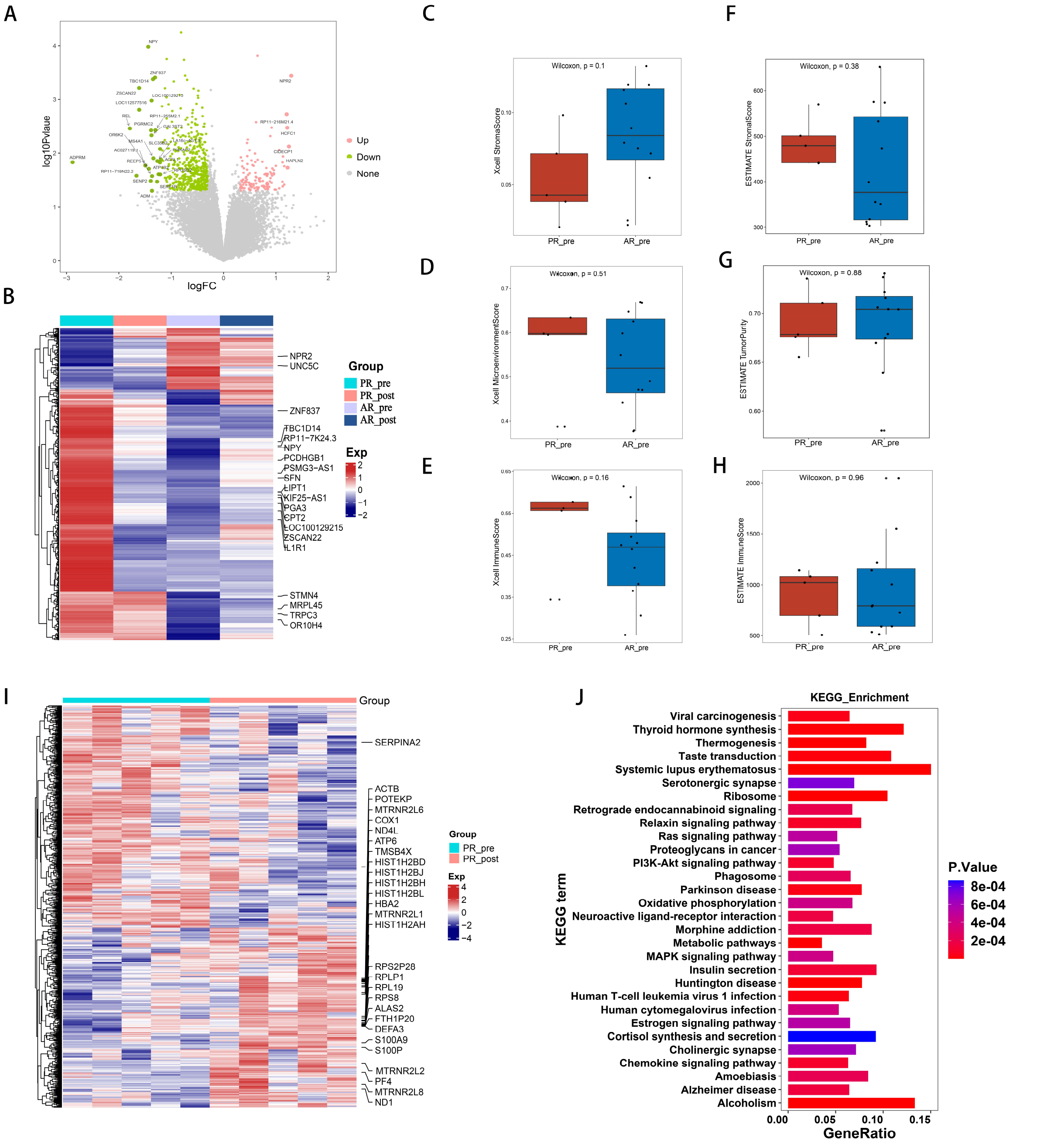

Supplementary Figure S3. (A) Volcano plots showing differences between PR_pre and AR_pre groups. (B) Heatmap analysis of mean expression of DEGs between PR_pre and AR_pre groups in four groups. (C-E) Boxplot showing xCell enrichment tumor purity, immune, and stromal scores between PR_pre and AR_pre groups. (F-H) Boxplot showing ESTIMATE enrichment scores of 14 stromal cells between PR_pre and AR_pre groups. (I) Heatmap analysis of DEGs between PR_pre and PR_post groups. (J) KEGG enrichment analysis of DEGs between PR_pre and PR_post groups.

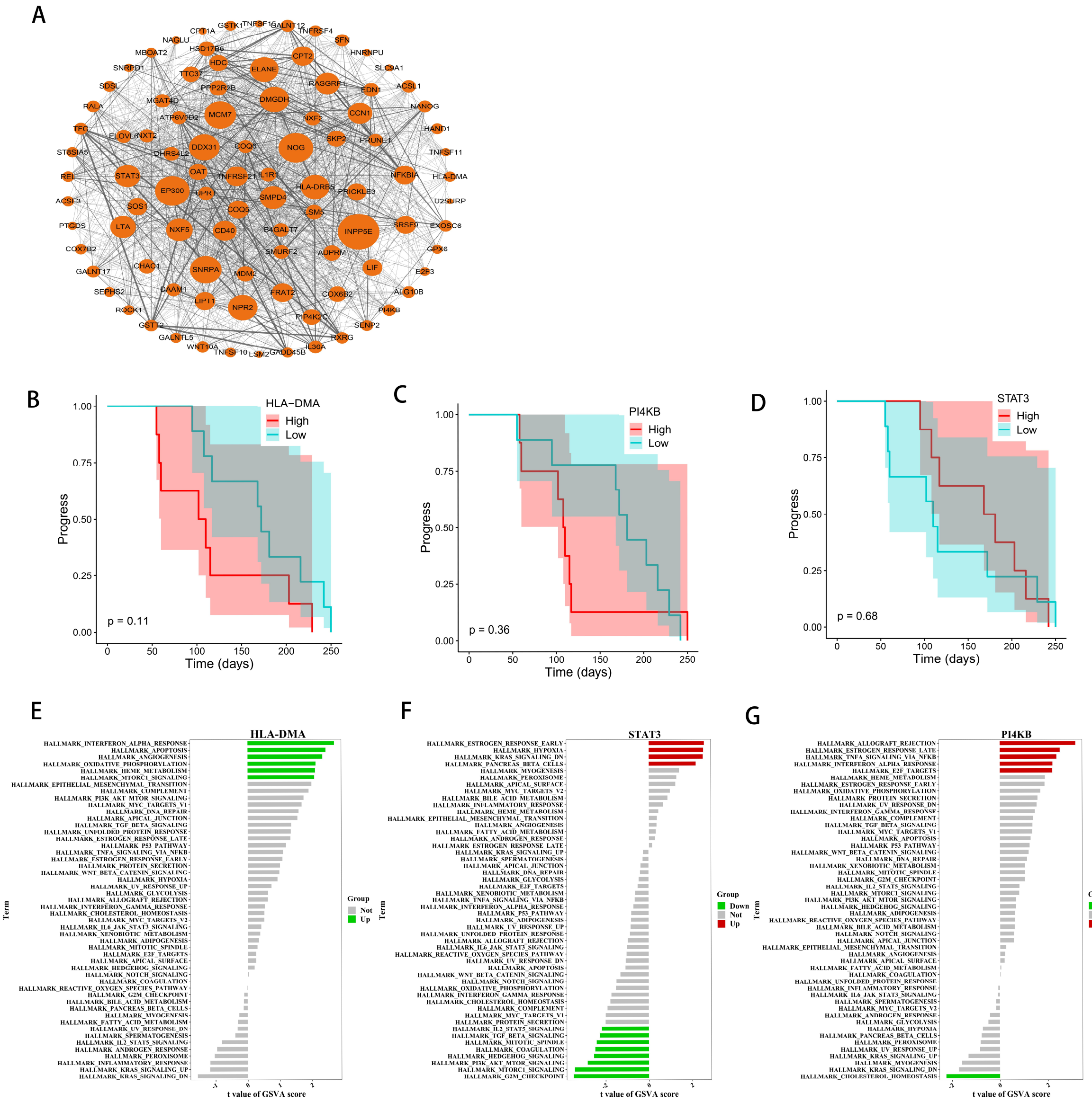

Supplementary Figure S4. (A) Network depicting co-expressed DEGs between PR_pre and AR_pre groups within cancer-associated KEGG pathways following WCGNA analysis. (B-D) Kaplan-Meier curve of *HLA-DMA*, *PI4KB* and *STAT3* with progression time. (E-G) GSVA of *HLA-DMA*, *PI4KB* and *STAT3*.

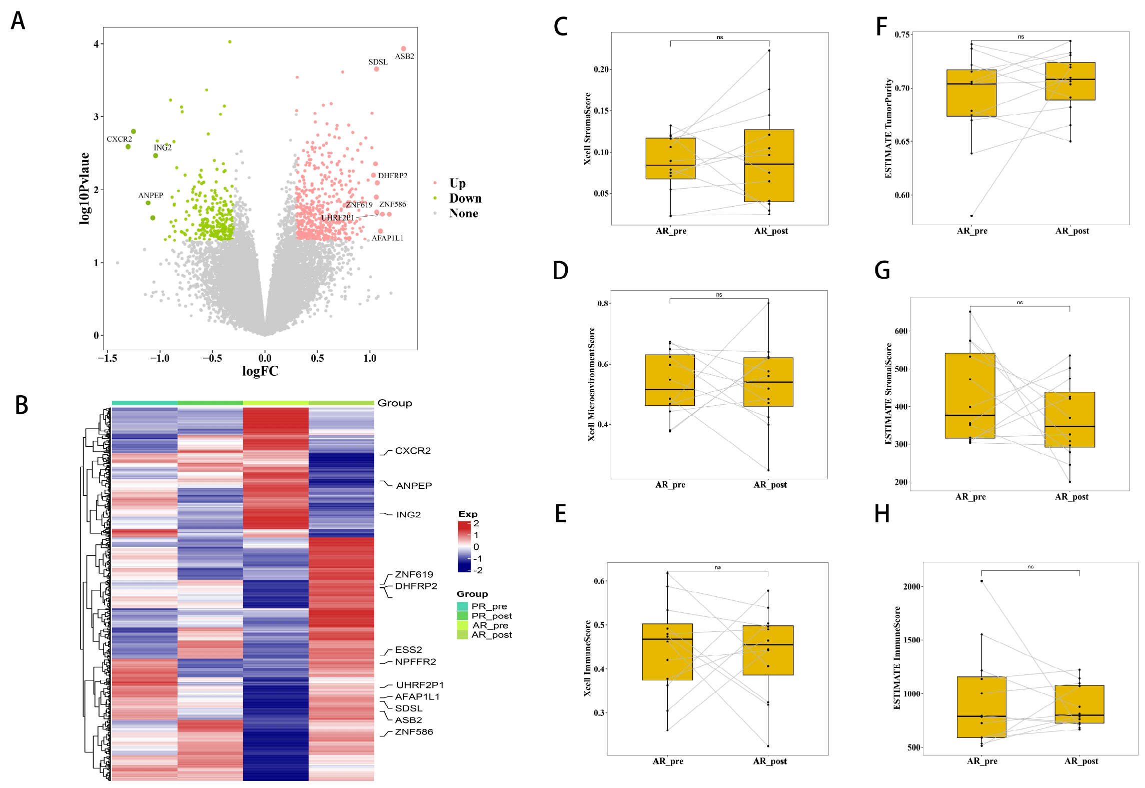

Supplementary Figure S5. (A) Volcano plots showing differences between AR_pre and AR_post groups. (B) Heatmap analysis of mean expression of DEGs between AR_pre and AR_post groups in four groups. (C-E) Boxplot showing xCell enrichment microenvironment, stromal, and immune scores between AR_pre and AR_post groups. (F-G) Boxplot showing ESTIMATE enrichment tumor purity, immune, and stromal scores between AR_pre and AR_post groups, where ns indicates not significant.

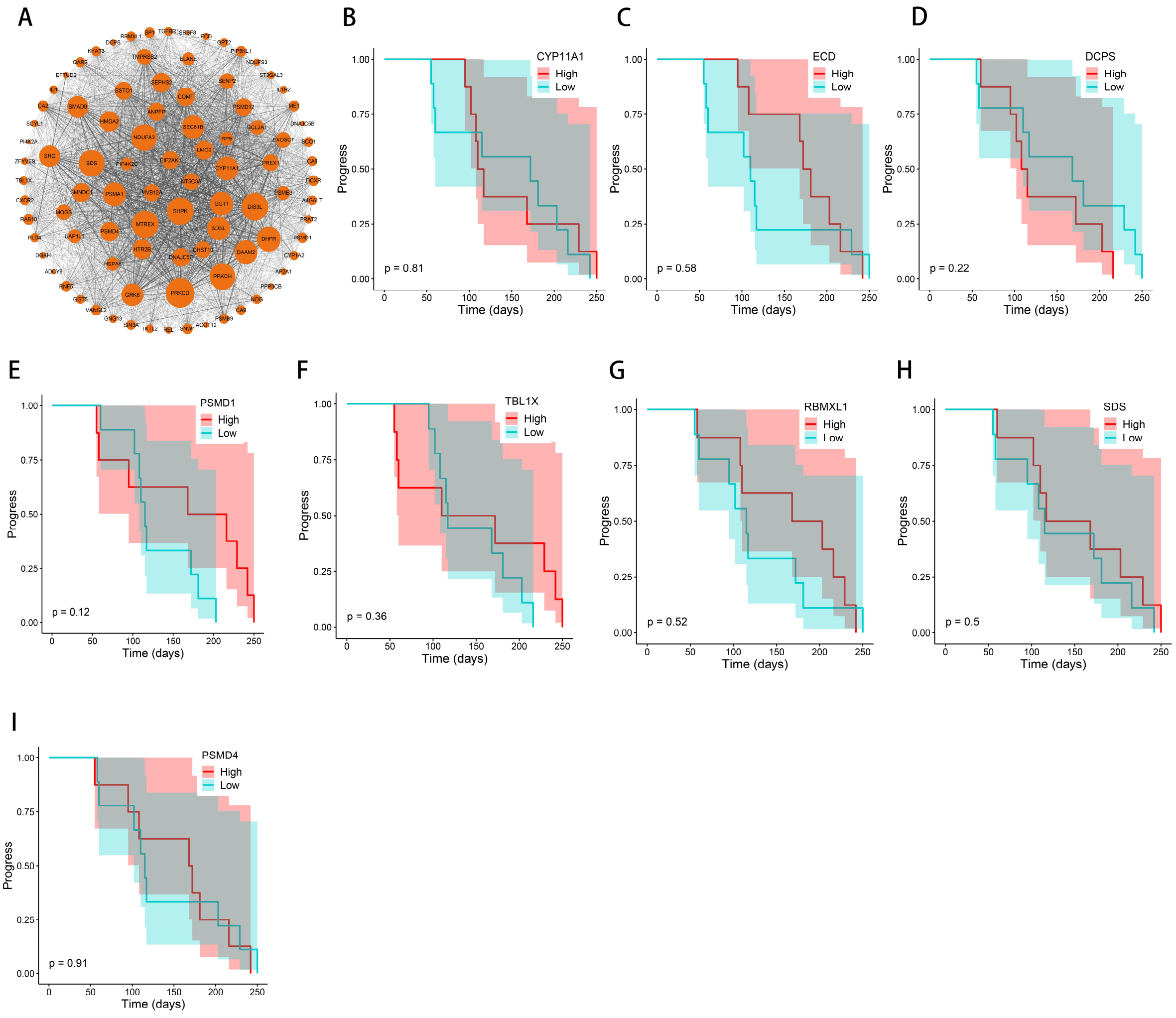

Supplementary Figure S6. (A) Network depicting co-expressed DEGs between AR_pre and AR_post groups within cancer-associated KEGG pathways following WCGNA analysis. (B-I) Kaplan-Meier curve of *CYP11A1*, *ECD*, *DCPS*, PSMD1, *TBL1X*, *PBMXL1*, *SDS*, and *PSMD4* with progression time.

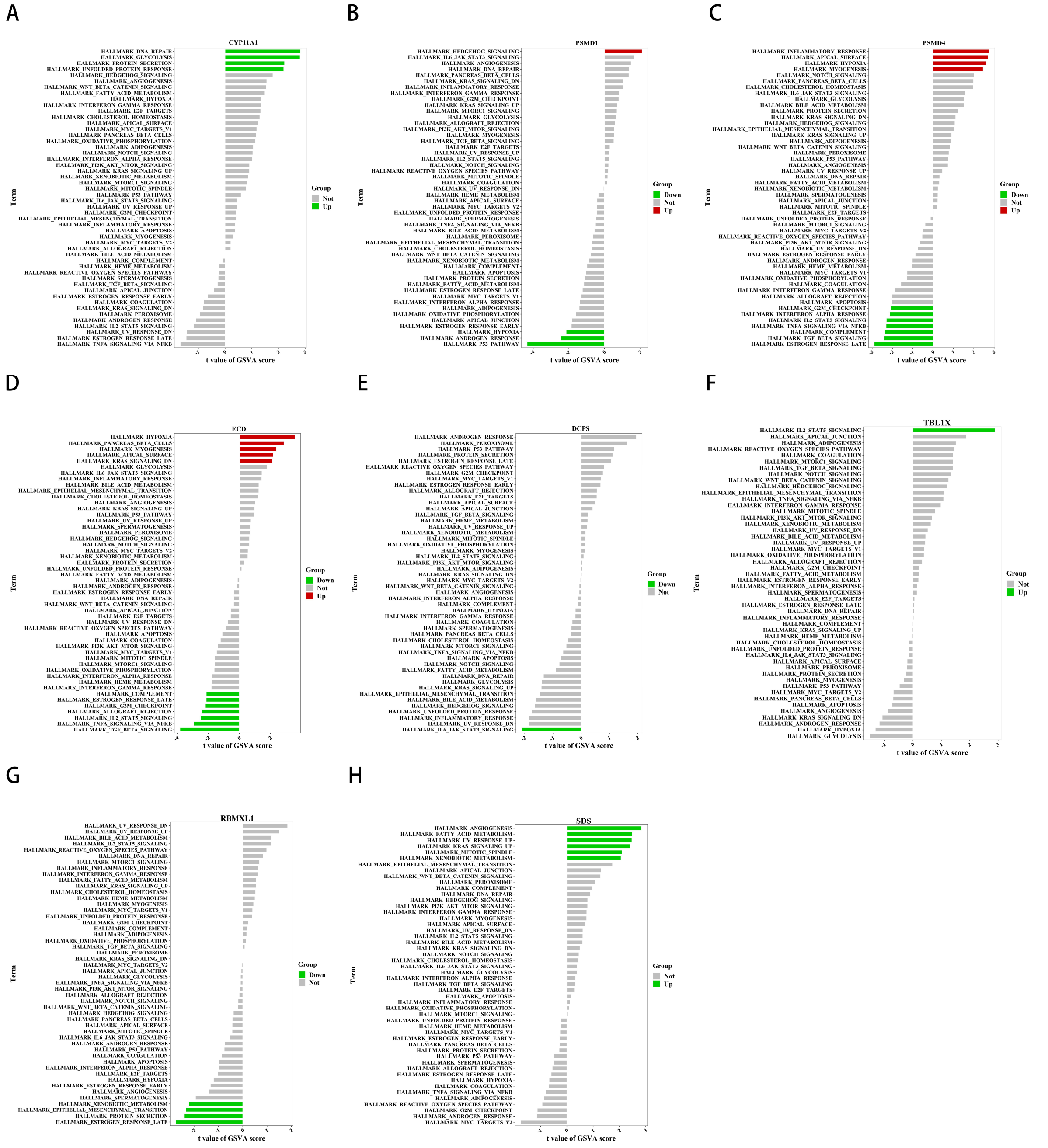

Supplementary Figure S7. (A-H) GSVA of *CYP11A1*, *ECD*, *DCPS*, PSMD1, *TBL1X*, *PBMXL1*, *SDS*, and *PSMD4*.
